# Rational Risk-Benefit Decision-Making in the Setting of Military Mefloquine Policy

**DOI:** 10.1155/2015/260106

**Published:** 2015-10-22

**Authors:** Remington L. Nevin

**Affiliations:** Department of Mental Health, Johns Hopkins Bloomberg School of Public Health, 624 N. Broadway, Room 782, Baltimore, MD 21205, USA

## Abstract

Mefloquine is an antimalarial drug that has been commonly used in military settings since its development by the US military in the late 1980s. Owing to the drug's neuropsychiatric contraindications and its high rate of inducing neuropsychiatric symptoms, which are contraindications to the drug's continued use, the routine prescribing of mefloquine in military settings may be problematic. Due to these considerations and to recent concerns of chronic and potentially permanent psychiatric and neurological sequelae arising from drug toxicity, military prescribing of mefloquine has recently decreased. In settings where mefloquine remains available, policies governing prescribing should reflect risk-benefit decision-making informed by the drug's perceived benefits and by consideration both of the risks identified in the drug's labeling and of specific military risks associated with its use. In this review, these risks are identified and recommendations are made for the rational prescribing of the drug in light of current evidence.

## 1. Introduction

The antimalarial drug mefloquine (commonly marketed as Lariam) had until recently enjoyed a long history of preferred use in certain military settings in the prophylaxis of chloroquine-resistant* P. falciparum* malaria. Originally developed by the US military in a Vietnam War-era drug development program and subsequently licensed for prophylactic use in the US in 1989 [1], mefloquine has, in the over quarter century since, been widely used by the US military and by various international militaries during deployments in malaria-endemic areas, including the Horn of Africa [2–4], sub-Saharan Africa [5], Australasia [6, 7], Southeast Asia [8], and the Middle East—particularly during recent large-scale operations in Iraq and Afghanistan [8, 9].

Mefloquine is a 4-quinolinemethanol closely related to quinine, and the drug shares a common structural core with related quinoline antimalarial and antiparasitic compounds that exhibit clinically significant but idiosyncratic neurotoxicity [10]. Recently, mefloquine was itself recognized as an idiosyncratic neurotoxicant that may cause permanent injury to the central nervous system (CNS) [10]. Mefloquine readily crosses the blood brain barrier (BBB), where it may adversely affect the function of neurons particularly in the limbic system and brainstem [10, 11]. In susceptible individuals, likely owing to idiosyncratic genetic and environmentally mediated variability in neuropharmacokinetics that may serve to limit its enzyme-mediated efflux back across the BBB [12], the drug may accumulate to intoxicating and even neurotoxic concentrations in the CNS during prophylactic use [10].

At prophylactic doses of 250 mg weekly, a sizeable minority of mefloquine users may experience one or more neuropsychiatric symptoms attributable to the drug [13]. Recent mefloquine drug labels describe “very common” side effects including insomnia and abnormal dreaming affecting greater than 10% of prophylactic users and “common” side effects including anxiety and depression affecting between 1 and 10% of prophylactic users [14]. Other side effects described as “uncommon” but reported in between 1 and 10 prophylactic users per 1,000 include agitation, aggression, restlessness, panic attacks, mood swings, and confusion [15].

Rather than representing isolated “side effects,” various mefloquine drug labels have emphasized that certain of these must be considered as “prodromal” symptoms [14, 16]—potentially indicating a personal idiosyncratic susceptibility to more serious CNS drug toxicity. For example, the current US drug label warns that “the occurrence of psychiatric symptoms such as acute anxiety, depression, restlessness or confusion suggest a risk for more serious psychiatric disturbances or neurologic adverse reactions” [17]. Among susceptible individuals, such prodromal symptoms may progress to a potentially life-threatening condition [11] that early drug labeling euphemistically referred to as “a more serious event” [16] but which may represent the effects of a progressive limbic encephalopathy caused by drug intoxication [18]. Such encephalopathy commonly manifests as a paranoid, manic, confusional, or dissociative psychosis and may be associated in severe cases with a risk of permanent neurological sequelae including neurotoxic brainstem injury [10, 18] and permanent psychiatric sequelae including homicidal violence and suicide [8, 11, 19, 20]. Among those reporting adverse events from the drug—and as would be expected from a postencephalopathy syndrome [11]—some continue to experience neurological and psychiatric sequelae, including vertigo, dizziness, disequilibrium [21], nightmare disorder, and cognitive impairment [22] years after acute intoxication. Such chronic neurological and psychiatric sequelae may plausibly confound the diagnosis and management of other conditions associated with military service, including traumatic brain injury and posttraumatic stress disorder [8, 23, 24].

Although drug label warnings dating back to the US introduction of mefloquine in 1989 noted the drug “must be discontinued” at the onset of certain listed prodromal symptoms [16], recently these warnings have been widely updated to more explicitly define under what conditions continued use of the drug should be contraindicated. In 2013, US drug regulators mandated the inclusion of a boxed warning on generic versions of the drug clarifying that “if psychiatric or neurologic symptoms occur” during prophylactic use—a warning cautiously encompassing all possible neuropsychiatric symptoms of the intoxication prodrome—the drug “should be discontinued” [17]. That same year, European regulators issued guidance to physicians that should those taking mefloquine experience “a neuropsychiatric reaction,” including “changes to their mental state,” “they should stop taking mefloquine immediately and seek urgent medical advice” [25]. Similarly, at least one “Dear Doctor” letter has explicitly counseled healthcare providers to advise their patients to “seek immediate medical advice” for any “psychological changes” including “sleep disorders” or “abnormal dreaming” that occur while taking the drug [26].

Prior to the these changes, the writings of numerous influential authors may have served to undermine earlier recommendations by the drug's manufacturer to immediately discontinue mefloquine at the onset of certain of these symptoms [27], by claiming, for example, that the drug could be continued even following the development of certain listed prodromal symptoms such as anxiety [28] or by suggesting that assumed prodromal symptoms of CNS toxicity might instead be due to causes other than mefloquine, such as recreational substance use, preexisting mental illness, the stresses of travel [28, 29], or stressful military environments [30]—failing to emphasize the recommendation to nonetheless immediately discontinue the drug despite this uncertainty.

Additionally, widespread confusion that only those with a history of certain mental health disorders were susceptible to the adverse psychiatric effects of mefloquine—a confusion which appears to have increased when such a history was added to the drug label as a formal contraindication [31]—may have contributed, at least in certain cases [32], to prodromal symptoms of CNS toxicity being erroneously misattributed to such a presumed history and not to the drug. In military settings, widespread belief among senior leaders that such prodromal symptoms occurred only rarely [33–35] and far less commonly than had been previously demonstrated [32] may have further contributed to the drug not being discontinued as often as drug label guidance recommended—for example, as rarely as in 1 in 334 users in one early example of US military use [4].

With recent strengthened warnings and with understanding that prodromal symptoms may occur quite commonly even in perfectly healthy individuals, efforts to better comply with drug label guidance now make the “very common” incidence of prodromal symptoms induced by mefloquine incompatible with the drug's widespread convenient and safe use in prophylaxis. In randomized blinded trials, at least 29% [36, 37] of prophylactic mefloquine users reported one or more neuropsychiatric symptoms consistent with the prodrome of intoxication, thus contraindicating continued use of the drug in accordance with current drug label guidance.

In military settings where the background incidence of such symptoms may already be increased owing to common stressors associated with deployment or to preexisting mental health conditions which may be prevalent in 10% or more of deploying military personnel [38], the recognition and correct attribution of prodromal symptoms to drug intoxication may be particularly confounded [23]. Such confounding increases the risk of potentially life-threatening or permanent sequelae of severe intoxication if the cause of such symptoms is erroneously misattributed and the drug is consequently not immediately discontinued.

As awareness has increased both of these considerations and of the potentially permanent effects of the drug's CNS toxicity [39–41], use of the drug has decreased [42, 43] and has been formally deprioritized by policy in certain military settings, in favor of the more widespread use of safer and better tolerated alternatives [44–46].

Although even the innovator license holder has conceded that mefloquine is no longer the most effective available antimalarial for the prevention of malaria [47], use of mefloquine in certain military settings has been claimed as potentially advantageous for a number of other reasons, including the drug's convenient weekly dosing (which may facilitate directly observed therapy) [48, 49], its somewhat lower cost relative to certain alternatives [50, 51], and its indication for long term use in the absence of contraindications [52].

Owing to slight international differences in such costs and indications, as well as to differences in risk-benefit decision-making, international military antimalarial policies have varied, even between deployments posing similar risks to military forces. For example, for military deployments to Djibouti (a nation located in the Horn of Africa) French military forces discontinued the widespread use of mefloquine in 2002 in favor of preferred use of the broad-spectrum antibiotic and antimalarial doxycycline [2]. In contrast, US forces, which had been prescribing mefloquine to its personnel there at a dose of 250 mg weekly, delayed preferred use of doxycycline until 2009 [2], before next substituting the combination drug atovaquone/proguanil (commonly marketed as Malarone) in 2011 [46]. In contrast, German forces have always preferentially used atovaquone/proguanil for the duration of their deployments there [2].

Military policies that retain the widespread use of mefloquine for its perceived advantages or for use in rare cases where preferred alternative antimalarials cannot be taken should reflect rational risk-benefit decision-making informed not only by awareness of the drug's presumed benefits and of the risks identified in the drug's labeling, but also by changing awareness, recognition, and acknowledgement of the risks associated with any use of the drug in military settings. In this review, specific risks associated with military use of mefloquine are identified, and recommendations for the rational use of the drug in military settings are then made in light of current evidence.

## 2. Military Specific Risks

Years prior to the original licensing of mefloquine, concerns were raised at certain risks associated with use of the drug in military settings. For example, one study, published in 1982, noted “legitimate concern for mefloquine's safe use in aircrewmen” [53]. Such initial concerns were motivated mostly by consideration of the drug's quinine-like neurological effects, which subsequently contributed to initial recommendations [54], later formalized, to prohibit all use of the drug among aviation personnel.

Although the initial US drug label had advised that owing to concerns of “neuropsychiatric reactions” “caution should be exercised” while “piloting airplanes,” “driving,” and “operating machines” [16], in contrast to concerns over its use in aviation personnel, with limited exceptions [55], these warnings did not contribute to particular recommendations against use of the drug in other military occupational settings—for example, among drivers or machine operators. Although early research would demonstrate that dizziness and “lightheadedness” were not infrequently reported [32], other research suggested, somewhat counterintuitively, that mefloquine either had no significant effect on [56] or could even improve psychomotor performance, including performance on certain driving tests [57]. Similarly, a study suggesting subjective “ability to work” was not affected by mefloquine may reflect the effects of selection bias owing to the study's very high nonresponse rate [58]. With recent understanding of the adverse neurological effects of mefloquine on structures in the brainstem, including the inferior olive [59], research has emerged demonstrating that mefloquine may impair motor learning during certain complex tasks, such as those comparable to marksmanship, with “clinical implications for mefloquine users” [60].

Similarly, although mefloquine was noted during early testing to adversely alter patterns of dreaming and significantly reduce overall sleep duration [32] and despite broad military acknowledgement of the importance of sleep hygiene [61], such concerns have not, until recently, significantly informed military use of the drug. Early warnings by the drug manufacturer for users of the drug to report “sleep disorders including abnormal dreaming” [26] to their physician were similarly not widely communicated. Only in recent years, as awareness has grown that symptoms of disturbed sleep, including insomnia and abnormal dreaming, are “very common” with prophylactic use of mefloquine [14] and that vivid nightmares described occasionally as having “technicolor clarity” [32] are not benign and should be considered contraindications to the drug's further use, have the potentially negative impact of these effects on military performance and military operations been more broadly considered in military settings.

Likewise, reports of symptoms such as panic attacks and confusion, which while being described as “uncommon” may nonetheless affect between 1 and 10 prophylactic users per 1,000 [15] and thus may not be infrequent during large military deployments, may be problematic in military settings. Disturbing case reports of deployed service members experiencing episodes of panic resulting in abnormal behavior [8] or of being confused and found “wandering aimlessly” [62] raise legitimate concerns for their likely occurrence in deployed environments. Of potentially similar concern are the drug's noted subclinical effects among military personnel particularly on measures of “tension” and “anger” [32], which may serve to measurably shift patterns of behavior in large populations exposed to the drug.

Although not unique to military settings, concern of suicide associated with use of the drug has also been a pervasive concern, particularly in the US military, dating at least back to the first large-scale deployment of troops to Iraq in 2003 during which use of mefloquine was widespread [8] and an increased risk of suicide was observed [63]. Although the US drug label had by this time acknowledged reports of suicide and suicidal ideation with the drug [64], military officials initially testified they did not believe that mefloquine “represents the big causal factor in our suicide rates” [33]. Yet subsequently, at least one military suicide was considered consistent on psychological autopsy with the effects of mefloquine intoxication [65]. A later media-affiliated study among Irish service members suggested that risk of suicide could have been increased up to fivefold among deployed cohorts exposed to mefloquine [66]. With mefloquine known to increase the risk of mental disorders [67] and with mental disorders known to increase risk of suicide [68], such results are plausible and qualitatively consistent with known suicide epidemiology. Unfortunately, despite independent recommendations [69] to more formally study the epidemiology of mefloquine suicide—and although such studies initially described as seeking “to dispel… [mefloquine] suicide myths” [70] had been previously claimed by senior military officials to be either planned or in progress [71, 72]—the results of these studies remain unpublished in the peer-reviewed literature. Recent large-scale military-sponsored studies of suicide have similarly failed to consider exposure to mefloquine in their risk factor analysis [73, 74], potentially confounding the observed association of suicide with deployments [73] where use of the drug may have been widespread [8, 75].

In addition to concerns of self-directed violence, mefloquine is strongly associated in postmarketing studies with risk of violence towards others, including homicide [20, 76], magnifying concerns of these consequences with the drug's use among military personnel who are likely to be heavily armed during use of the drug. Owing to the known association of the drug with agitation and aggression, which may affect between 1 and 10 in 1000 users [15], and to the drug's known association with symptoms of more acute intoxication including dissociative and paranoid psychosis, there has been reasonable speculation by military and government officials that mefloquine intoxication may have contributed to cases of homicidal violence overseas [77] and among returned service members [78].

Similar concerns arose recently following receipt by the US Food and Drug Administration (FDA) of a drug adverse event report of uncertain provenance [79] describing a US service member who “developed homicidal behavior” which “led to [h]omicide killing 17 Afghanis [sic].” Although this report alludes clearly to a well-known case of a soldier found guilty of a similar crime who had been issued with mefloquine during a prior deployment [80], US military officials have neither confirmed nor denied his use of mefloquine during his most recent deployment [81], making any causal association of this incident with acute intoxication speculative.

Intriguingly, although public records reveal the soldier had been prescribed doxycycline, at the time of his arrest, this bottle of medication was found sealed and unopened [82]. At the time of the incident, the soldier was assigned to a special forces unit affiliated with US Army Special Operations Command (USASOC) and was known to have been given multiple prescription drugs without documentation by special forces personnel [82, 83]. Within a year and a half of the massacre, USASOC issued a formal order prohibiting the use of mefloquine among its personnel, acknowledging that “consideration must be made for the impact of this medication on our population” [44].

## 3. Discussion

In the over quarter century of international military use of mefloquine, many of the drug's unique risks in military settings have become more widely appreciated and now more routinely inform policies for the use of the drug. Similarly, over this period, many of the drug's perceived advantages have been disproved both by formal evidence and by experience.

For example, and in contrast to original expectations, certain studies have found equal or higher compliance with daily as compared with weekly prophylaxis [84, 85], and recent military deployments where daily antimalarial drugs have been prioritized for use have been ecologically associated with significantly lower rates of malaria than comparable deployments where mefloquine had previously been the drug of choice [5, 86, 87]. A number of these daily antimalarial drugs have also obtained formal indications for use in many jurisdictions independent of duration of therapy [88, 89], removing one of the remaining perceived advantages of mefloquine [58] relative to these safer and better tolerated medications.

Likewise, the findings of prior economic analyses which have found cost advantages with use of mefloquine [50, 52, 90] are typically not generalizable to military settings, in that these analyses fail to consider the high potential costs of risks unique to these contexts. As experience and this review have demonstrated, these costs, which include both direct and indirect economic costs, may be significant.

As described elsewhere [13], even independent of these considerations—and although mefloquine clearly remains indicated for prophylaxis in every jurisdiction in which it was originally licensed—owing to the high rate of preexisting contraindications to its use and to the high rate of induced contraindications with use of the drug, convenient and safe use of mefloquine on a widespread basis as a “drug of choice” is now prohibitive in most military settings. While such considerations alone should preclude the mass prescribing of mefloquine as a first-line agent, use of the drug may still be considered by some militaries as a second- or third-line agent [55], in spite of the broader concerns identified in this review, on an individualized basis among those with contraindications or intolerance to preferred alternative antimalarials.

Unlike the case with mefloquine, true contraindications to alternative drugs for prophylaxis of chloroquine-resistant* P. falciparum* are very rare: of the alternative drugs commonly available internationally for this indication, although intolerance to doxycycline is not at all uncommon [91], atovaquone/proguanil is, in contrast, exceptionally well tolerated [85, 88], with blinded trials reporting a rate of discontinuation due to adverse events of only 1% to 2% during prophylactic use [36, 37]. Military policies that deprioritize use of mefloquine to a second- or third-line drug, for use only in those with contraindications or intolerance to these alternatives, should therefore expect to see fewer than 1-2% prescribed mefloquine, and any greater rate of prescribing should prompt a careful review of prescribing practices [75] to identify the causes of deviation from such policy. Additionally, certain recommendations, first described elsewhere [13] and outlined more fully below, should be considered in the setting of military policies that permit continued use of the drug.

## 4. Recommendations

Military policies that permit continued use of mefloquine as a second- or third-line antimalarial drug should ensure the implementation of a number of precautions to properly comply with recent labeling guidance and to reduce the risk of more severe intoxication and its potentially chronic, permanent, or life-threatening sequelae.

First, in accordance with international labeling guidance, such policies must ensure that service members that prescribed mefloquine are informed that any neuropsychiatric symptoms that may develop while taking the drug may be evidence of a personal susceptibility to drug intoxication that should mandate its immediate discontinuation. Although prior to recent labeling changes such symptoms were poorly appreciated as evidence of CNS toxicity and commonly attributed to other causes, current drug label guidance has clarified that even relatively common symptoms, including insomnia or other sleep disturbances, vivid dreams or nightmares, mild anxiety or depressive symptoms, and other even potentially subtle changes in “mental state” such as irritability or personality change, should be considered as cause to seek medical attention and immediately discontinue the drug [13].

Similarly, counseling at the time of prescribing and dispensing should extend beyond the mere issuance of a printed warning (or “wallet card”) and be complemented by a documented test of knowledge of its contents, as well as by educational efforts extended throughout the service member's chain of command, particularly to ensure that others are aware of the typically subtle signs and symptoms of mefloquine intoxication. In prior military settings, absent widespread awareness of the symptoms of intoxication, these have been both occasionally overlooked or unrecognized by the service member [18, 62] or even when recognized they were erroneously attributed both by medical personnel and to the chain of command to causes other than the drug [8, 13].

Second, particularly in military settings where strong disincentives may exist against the reporting of mental health symptoms, including those resulting from fears of stigma, even with adequate education, certain intoxicated patients may fail to heed drug label guidance to report such symptoms and may therefore risk continuing taking the drug. To minimize these risks, where directly observed therapy is implemented, this should be conducted in private by medical personnel and not through the chain of command so as to minimize barriers to reporting of potentially stigmatizing prodromal symptoms. Similarly, where directly observed therapy is not implemented, medical personnel should nonetheless conduct routine evaluations of those on the drug to rule out prodromal symptoms of intoxication—such as paranoia or confusion—which may limit such reporting [13, 80, 92].

Third, as many cases of mild intoxication—though not all—may be identified during the first few weeks of drug use [27], to further reduce the risk of more severe intoxication with continued dosing, military policies should strongly consider limiting initial prescribing of the drug to a small number of tablets to be taken prior to deployment, with the service member evaluated regularly and carefully by medical personnel during this period to assess the development of prodromal symptoms. If none are detected, policy could then permit prescribing the remaining tablets for deployment [13].

Similarly, as it can take as many as 7–10 weekly doses of mefloquine for the drug to achieve steady state and protective concentrations in serum, where deployment dates are known this far in advance, a prolonged period of use prior to deployment should be considered both to improve the drug's antimalarial effectiveness and to further minimize the risk of unrecognized intoxication that might occur during deployment. This consideration is particularly relevant during remote deployments, where the patient may be far from medical care and where certain of the preceding recommendations requiring medical evaluation may not be feasible [13].

Finally, and in this respect, the military clinician and the chain of command should be prepared for the consequences of the need for service members to immediately discontinue the medication while in a malaria-endemic area and when far from medical care. In those cases where the drug is prescribed as a second-line drug, policy should require the coprescribing of a few weeks' supply of an alternative third-line antimalarial, to be used on discontinuation of mefloquine until medical evaluation can be arranged. Similarly, in those rare cases where the drug is prescribed as a “drug of last resort”—as such use implies that no other prophylactic medications are available to switch to—in areas where malaria is highly endemic and where mosquito-avoidance measures alone may be insufficient, this may mandate the service member's early evacuation to minimize risks when mefloquine is discontinued. Although under such conditions it may appear reasonable for the chain of command or the military clinician to recommend continuing the use of mefloquine until evacuation can be arranged, the risks associated with such continued dosing could outweigh even the risk of sequelae from a treatable episode of malaria that could develop during the period, making such a recommendation decidedly unwise [13].

Faithful implementation of these recommendations may serve to minimize the risks associated with use of mefloquine. However, given that severe intoxication and permanent effects have been reported after as little as a single 250 mg tablet [93], these recommendations may serve to minimize but will not fully eliminate the unique military risks considered in this review that are associated with continued use of mefloquine, even rarely as a second- or third-line drug.

## 5. Conclusions

Military policies that permit the continued use of mefloquine expose militaries to certain unique risks not encountered with most civilian use of the drug. These risks, when fully recognized and acknowledged, exceed the drug's benefits in many military settings. While international drug regulators may consider a more limited set of risks when addressing issues in drug safety regulation, militaries must consider these additional risks in formulating policies for the rational use of this medication. Consideration of the issues in this review may aid militaries in formulating rational policies for the safer use of the drug. Depending on these militaries' risk tolerance, such consideration may serve to motivate further prohibitions on the use of mefloquine in line with those already in place in a growing number of military settings.
